# Accessible Patient Education Materials for Low Back Pain Rarely Meet People's Information Needs: A Scoping Review

**DOI:** 10.1002/msc.70130

**Published:** 2025-05-30

**Authors:** Chloé Debonne, Axel Houdart, Chloé Cachinho, Alexy Ouvrier‐Neyret, Thomas Gérard, Valentin Vaillant, Yannick Tousignant‐Laflamme, Marie‐Pierre Gagnon, Maxime Sasseville, Simon Décary, Florian Naye

**Affiliations:** ^1^ CHU de Toulouse—Antenne IFMK Rodez CCI de l’Aveyron Rodez France; ^2^ Institut de Formation et de Recherche En Santé (IFRES) Alençon France; ^3^ Faculty of Medicine and Health Sciences Université de Sherbrooke School of Rehabilitation Research Centre of the CHUS CIUSSS de l’Estrie‐CHUS Sherbrooke Canada; ^4^ Maison de santé du Taillefer Duingt France; ^5^ Institut de Kinésithérapie Podologie Orthopédie (IKPO) de la région sanitaire de Lille Lille France; ^6^ Faculté des sciences infirmières Université Laval Québec Canada

**Keywords:** health equity, information needs, low back pain, patient education, stakeholder involvement

## Abstract

**Background:**

Patient education is a cornerstone of care for individuals with non‐specific low back pain (LBP). However, little is known about whether accessible patient education materials (PEMs) meet people's information needs.

**Methods:**

We conducted a scoping review following the JBI methodology and reported results according to PRISMA‐ScR. We systematically reviewed three databases: Ovid MEDLINE, Scopus, and CINAHL. The search strategy was iteratively developed and peer‐reviewed using the PRESS checklist. Eligible studies had to provide full access to the PEM designed for people with LBP. Study selection and data extraction were performed independently and in duplicate. Five reviewers conducted a consensus‐based analysis by independently matching PEM content to eight categories of information needs derived from previous research.

**Results:**

Of 9617 citations identified, 23 studies met inclusion criteria, yielding 41 unique PEMs. We excluded many citations (67.3%) because the PEM used in the study was missing. Most PEMs were in English (95%) and took the form of posters, booklets, or leaflets. Only eight PEMs (19.5%) reported readability assessment. Stakeholder involvement was reported in eight studies. Among PEMs with stakeholder input, characteristics from the PROGRESS + framework were rarely disclosed. Only one PEM addressed all eight identified information needs. The most frequently covered information needs were treatment options (65.9%) and imaging (61.0%), while information on prognosis and flare management was scarce (17.1%).

**Conclusion:**

Accessible PEMs for non‐specific LBP rarely meet the full spectrum of patient information needs. Improving stakeholder involvement and readability assessment is essential to enhance the usefulness and equity of educational resources.

## 1 Introduction

Patient education is a fundamental component of care for individuals with non‐specific low back pain. It is defined as a structured process aimed at influencing patient behaviour by fostering the knowledge, attitudes, and skills necessary to support or improve health (Rankin et al. 2004). Evidence from recent meta‐analyses highlights the benefits of patient education, particularly for individuals living with chronic low back pain (Furlong et al. 2022; Ma et al. 2024; Piano et al. 2025). Accordingly, clinical guidelines recommend the inclusion of patient education in the management of non‐specific low back pain (Zaina et al. 2023). Despite this recommendation, the implementation of patient education remains suboptimal (J. Zadro et al. 2019), underscoring the need for effective implementation strategies (Liang et al. 2017). One such strategy involves improving the accessibility of patient education materials (PEMs) to clinicians. Ensuring that these materials are freely available— preferably as supplementary files accompanying peer‐reviewed articles—could help reduce practical barriers and support their integration into routine clinical practice.

PEMs are designed to convey information about diagnosis, prognosis, and pain management to individuals living with low back pain (Furlong et al. 2022). Their objectives include reshaping unhelpful beliefs about pain and their condition, providing reassurance regarding the evolution (i.e., prognosis), and shaping realistic recovery expectations (Furlong et al. 2022). PEMs can take various forms, ranging from printed booklets to interactive digital platforms. However, a recent meta‐analysis found that one‐size‐fits‐all educational interventions are largely ineffective for this population (Gomes et al. 2024), highlighting the need to tailor educational content to individual's needs. A scoping review clarified the concept of pain neuroscience education and described its core content, which focuses primarily on reconceptualising pain through a neurobiological lens (Adenis et al. 2023). However, this perspective does not address other information needs commonly sought by people living with low back pain, such as diagnosis, prognosis, treatment options, and support services (Lim et al. 2019). These findings suggest that for patient education materials to be truly effective, they must not only convey accurate information but also be responsive to the full spectrum of information needs of this population.

Equitable, inclusive, and culturally responsive PEMs are essential to effectively meet the needs of diverse populations. Culture significantly influences how individuals interpret and respond to health information (Chachkes and Christ 1996; Reis et al. 2022). It shapes beliefs about pain—including its causes, meanings, and coping strategies—which, in turn, affect how individuals perceive their condition and choose among treatment options (Chachkes and Christ 1996). For example, while some cultural groups view pain as a natural aspect of life, others may attribute it to spiritual or moral factors, such as punishment, leading to different understandings and treatment preferences (Paley et al. 2023). While cultural factors shape the meaning and interpretation of pain, patients' ability to comprehend and act on health information is also influenced by health literacy and education level. Systematic reviews have shown that many of these materials are written above the recommended sixth‐grade reading level, reducing their accessibility for individuals with limited literacy or lower educational attainment (Boutemen and Miller 2023; Rooney et al. 2021). These limitations can prevent people from fully understanding or engaging with the information provided. To mitigate these inequities, it is crucial to involve individuals from diverse backgrounds and with lived experience in the development of PEMs. Such collaboration can help ensure that the content is both culturally relevant and accessible to a broader audience.

The objectives of this scoping review were to (1) map the content of patient education materials accessible in the scientific literature for people living with non‐specific low back pain, (2) identify gaps between the content of these education materials and the information needs of this population, and (3) to report information on the accessibility, equity, diversity, and inclusivity of these materials.

## 2 Methods

### 2.1 Research Questions

This scoping review addressed the overarching question: Do accessible PEMs meet the information needs of individuals living with non‐specific low back pain? The specific questions were:

– What is the content of the accessible PEMs for people living with non‐specific low back pain?
– Are people living with non‐specific low back pain and other stakeholders involved in the development and/or pilot testing of these PEMs? What are the characteristics of equity, diversity and inclusion reported in this context?
– Do the accessible PEMs assess for readability?
– What are the gaps between the content of the accessible PEMs and the information needs of people living with non‐specific low back pain?

### 2.2 Patient and Public Involvement

In accordance with the Strategy for Patient‐Oriented Research and Patient Engagement framework of the Canadian Institutes of Health Research (Canadian Institutes of Health Research 2014), patients (CC, FN) and clinician partners (AH, AON) were members of the team. All partners involved in this scoping review were from urban areas of high‐income countries, identified as white, and shared a Western cultural background. All had a university‐level education or equivalent and demonstrated a high level of health literacy. Two were migrants with precarious status, and one partner was living with a physical disability. One partner also worked professionally with populations from migration backgrounds. They previously identified the tasks which were most meaningful for them (e.g., selection process, interpretation of the results). This ensured that patients and clinicians' needs, experiences and perspectives were incorporated in the study design, analysis, interpretation and dissemination (Canadian Institutes of Health Research 2014; Hoekstra et al. 2020).

### 2.3 Study Design

We conducted a scoping review following the current update of the Joanna Briggs Institute guidance (Peters et al. 2022). We reported the findings according to the Preferred Reporting Items for Systematic Reviews and Meta‐Analyses Extension for Scoping Reviews (PRISMA‐ScR) (Tricco et al. 2018) and registered the protocol in the Open Science Framework (doi: 10.17605/OSF.IO/M597B).

### 2.4 Eligibility Criteria

The eligibility criteria for the research questions according to the Population Concept and Context (PCC) framework are presented in Table 1.

**TABLE 1:** Study eligibility criteria.

Category	Study eligibility criteria
Population	Adults living with non‐specific low back pain
Concept	Patient education materials (e.g., pamphlets, booklets, posters, websites, videos, mobile applications, e‐learning, infographics) To be eligible, studies had to provide explicit access to the patient education material used—that is, the material had to be available within the article itself Exclusion: Patient education materials originating from social media or general online sources, and not developed for research purposes, were excluded due to potential concerns about their methodological rigour and content quality (Sridhar and King 2025)
Context	Any clinical or research contexts
Study designs	Any primary studies reporting the development or use of a PEM (e.g., randomised trials, mixed methods, protocols, development and pilot testing). We excluded conference abstracts, literature reviews, editorials, thesis and consensus statements, letters, and commentaries.
Language of publication	English or French

### 2.5 Literature Search Strategy

We developed the search strategy using an iterative process. A PhD candidate with training in search strategy peer‐reviewed the MEDLINE strategy, using the Peer Review of Electronic Search Strategies (PRESS) checklist (McGowan et al. 2016). We tested the final search strategy for sensitivity with five relevant articles suggested by the team review. Strategies utilised the PubMed thesaurus to operationalise the population and concept of our PCC framework. We adjusted vocabulary and syntax across databases. There were no date restrictions. We provide specific details regarding the search strategies for each database in Supporting Information S1: Appendix 1.

We performed a systematic search using multiple sources, including the Ovid MEDLINE, CINAHL (Ebsco platform) and Scopus databases. All searches were performed on August, 15^th^ 2024 from inception to that date.

### 2.6 Study Selection

**FIGURE 1:**
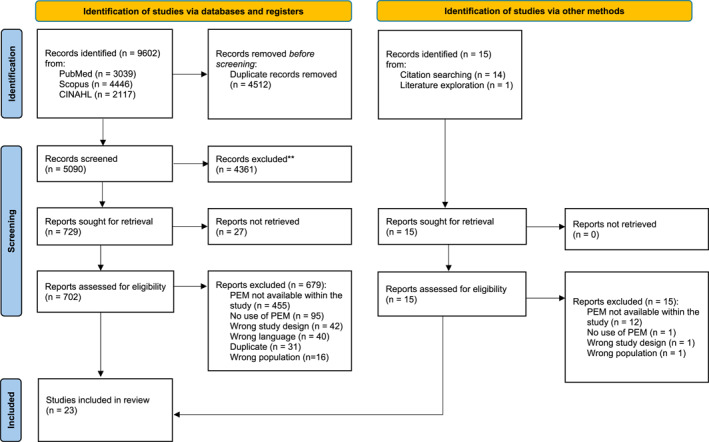
PRISMA flowchart. PEM, patient education material.

We used the systematic review management software Rayyan software for study selection (Ouzzani et al. 2016). We conducted a two‐step calibration training of the screening: (1) we collectively reviewed 10 references identified with the search strategy with a think‐aloud approach to align and clarify the reviewers' cognitive process (Wright et al. 2021), and (2) we independently reviewed 30 references to calibrate eligibility criteria interpretation between reviewers. If inter‐rater agreements (percent agreement) were below 80%, we clarified the eligibility criteria and conducted a new calibration training on 30 references. Pairs of reviewers (AH, AON, CC, CD, FN) performed an independent title and abstract and then a full text selection process. As FN had methodological expertise in conducting scoping reviews, he screened all citations and potential studies. We handled disagreements between reviewers by reaching a consensus of the review team. We conducted a structured handsearching of primary studies of all the included studies and recent literature reviews (from 2022 to 2024) identified during the selection process with backward and forward citations on Scopus (Briscoe et al. 2020). This ensured the rigorous coverage of the literature. We documented reasons for full text exclusion and reported them using the PRISMA 2020 flow chart in Figure 1.

### 2.7 Data Extraction

The research team codeveloped the extraction grid. We conducted a calibration training on four included studies to ensure a mutual understanding of the variables to extract and to ensure that the form adequately captures the desired information. Pairs of reviewers (AH, AON, CC, CD, FN) independently extracted the data. We resolved disagreements by team consensus. Extracted variables are presented in Table 2.

**TABLE 2:** Extracted variables.

Category	Extracted variables
Characteristics of the study	First author Year of publication Country of recruitment Objectives of the study Tasks required of participants when involved
Characteristics of the sample recruited for the development and/or pilot testing of the patient education material	Sample size Low back pain sub‐group (i.e., acute, subacute, and/or chronic) Age Gender Health literacy Presence of any variables of the PROGRESS + framework (O'Neill et al. 2014)
Characteristics of the patient education material	Format (e.g., booklet, poster, video) Language Content included Reported readability level Barriers for clinical integration (e.g., number of pages, time of the video, material available in a payable access article)

### 2.8 Data Synthesis

We conducted descriptive and tabular analyses for all objectives. For the format of the PEMs, we used the following categorisations: a leaflet was defined as a printed, double‐sided sheet folded to form multiple sections. A booklet is a short publication consisting of several bound pages. A slideshow refers to a visual presentation composed of multiple sequential slides. A website format describes an online resource accessible through a unique link that potentially comprises multiple web pages. A video is an animated visually recording, frequently accompanied by audio. A poster refers to a large, single‐sided infographic page. Finally, we classified regularly emailed informational bulletins under newsletter.

For Objective 2, our analysis was based on the information needs previously identified in a systematic review (Lim et al. 2019). This review identified 11 distinct information needs, clustered to facilitate synthesis. We grouped ‘general information regarding LBP management’, ‘tailored information regarding LBP management’, and ‘information regarding pain management’ into a single category. This process resulted in eight overarching categories: (1) general information related to low back pain, (2) diagnosis and cause, (3) needs for imaging, (4) prognosis, (5) flares and preventive measures, (6) low back pain management, (7) self‐management strategies, and (8) support services. To ensure analytical rigour, five raters (AH, AON, CC, CD, FN) conducted a consensus‐based approach by independently matching the content of the educational materials to the identified information needs. Consensus was defined as at least four out of five raters providing the same response for a given information need. In cases where only two or three raters provided the same response, we resolved dissensus through a team meeting.

## 3 Results

### 3.1 Study Selection

We present the PRISMA‐2020 flow chart of the selection process in Figure 1. We retrieved a total of 9602 citations from the search strategy. After removing duplicates, 5090 citations remained for the selection process. Of these citations, 729 citations were retained for full text screening, and 23 were included for data extraction. An additional 15 records were identified through citation searching and literature exploration; none were included. We excluded many citations in the full text screening (*n* = 467, 67.3%) because the patient education material used in the study was missing.

### 3.2 Characteristics of the Included Studies

The characteristics of the 23 included studies are presented in Table 3. Most studies were conducted in Australia (*n* = 11, 47.8%), followed by Brazil (*n* = 2, 8.7%), France (*n* = 2, 8.7%), the United Kingdom (*n* = 2, 8.7%) and the United States of America (*n* = 2, 8.7%). One study (4.3%) reported multiple recruitment sites. The included studies were published between 2014 and 2024, with 17 (73.9%) since 2020. Eight studies (34.8%) specifically addressed chronic LBP, one (4.3%) all LBP categories (i.e., acute, subacute and chronic), while seven (30.4%) did not provide this specific information.

**TABLE 3:** Characteristics of the included studies.

Authors (year of publication)	Country where the study was conducted	Study objectives and participant involvement	LBP categories	Sample size and participant characteristics for material development or pilot testing	Name of the available patient education material
Tomás‐Rodríguez et al. (2024)	Spain	*Study objective* To explore the effect of a 60 min single session of pain neuroscience education as an adjunct to back school on pain intensity and psychological variables patients with chronic low back pain. *No participant involvement*	Chronic	No information	Understanding and coping with pain
Marins et al. (2023)	Brazil	*Study objective* To evaluate the effectiveness of a smartphone app‐based self‐managed exercise programme plus health education, compared to a health education programme alone, on neuromuscular and perceptual outcomes in police officers and firefighters with chronic non‐specific low back pain. *No participant involvement*	Chronic	No information	Back book
Marley et al. (2023)	Lithuania, Northern Ireland, Italy, Sweden, and Portugal	*Study objective* To test the usability, acceptability and feasibility of an evidence‐based, digital education programme for people living and working with persistent LBP who are in sedentary or physically demanding jobs and need advice on ergonomics, self‐management of pain and healthy behavioural strategies. *No participant involvement*	Chronic	No information	MyRelief
Marcelo et al. (2023)	Australia	*Study objectives* (1) develop two educational consumer leaflets to support opioid tapering in older people with low back pain and hip or knee osteoarthritis, and (2) evaluate the perceived usability, acceptability, and credibility of the consumer leaflets from the perspective of consumers and healthcare professionals. *Participant involvement* To review the leaflet prototypes developed by the team (researchers and clinicians).	No information	*Consumers* *n* = 30, including 28 people living with low back pain and/or hip or knee osteoarthritis and 2 carers 17 women (56.7%) Mean age: 73.4 (SD = 5.7) Information on health literacy level, education level, and opioid consumption *Healthcare professionals* *n* = 20, including 9 physiotherapists, 7 pharmacists, 1 orthopaedic surgeon, 1 rheumatologist, 1 nurse practitioner, and 1 general practitioner	Stopping my opioids (pain medicine)
Agre and Agrawal (2023)	India	*Study objective* To compare the effect of ‘At‐Job’ exercises and ergonomic brochures on pain and disability in preprimary school teachers suffering from low back pain, since both can be administered at work. *No participant involvement*	Chronic	No information	Ergonomic brochure: Instructions for daily routine
Patterson et al. (2022)	Australia	*Study objective* To determine the feasibility of a patient‐education booklet to support patients with low back pain to reduce paracetamol intake. *No participant involvement*	Acute and chronic	No information	Know what you are taking
Webber et al. (2022)	United Kingdom	*Study objective* To use creative co‐design to produce prototype evidence‐based back pain educational resources that were sensitive to context. *Participant involvement* To co‐design the content through creative workshops, generate ideas using visual methods, refine and prioritise concepts, and review and test prototypes iteratively.	No information	*People living with low back pain* *n* = 8 *Healthcare professionals* *n* = 7	How can back pain affect you?
J. R. Zadro et al. (2022)	Australia	*Study objective* This study describes the protocol for a randomised controlled trial that aims to establish the feasibility of delivering and evaluating stratified care integrated with telehealth (‘rapid stratified telehealth’), which aims to reduce waiting times for low back pain. *No participant involvement*	No information	No information	Managing low back pain
Diniz et al. (2022)	Brazil	*Study objective* To investigate what format for providing patient information (i.e. written summary, infographic or video animation) is most effective for promoting correct beliefs about imaging and inevitable consequences of low back pain. *Participant involvement* To check whether the materials were easily understandable.	Acute, subacute, and chronic	*People living with low back pain* *n* = 15	*Four PEMs*: Management of low back pain ‐ right scenario versus wrong scenario Low back pain: Assessment and imaging Low back pain ‐ diagnosis and treatment Low back pain: General treatments
Simula et al. (2021)	Finland	*Study objective* To assess the effectiveness of this booklet as part of primary care for low back pain patients in comparison to usual care. *No participant involvement*	Acute	No information	Understanding my low back pain and whether I need imaging
Sharma, Traeger, Tcharkhedian, et al. (2021)	Australia	*Study objective* To evaluate community responses to a public health campaign designed for health service waiting rooms that focuses on the harms of unnecessary diagnostic imaging for low back pain. *Participant involvement* To evaluate the clarity and acceptability of messages and materials.	No information	*People living with low back pain* *n* = 19 12 women (63.2%) Age: 3 categories from 20 to 79 Information on education level, health literacy level, and country of birth	*Seven PEMs*: Sharma's poster #1 Sharma's poster #2 Sharma's poster #3 Sharma's poster #4 Sharma's poster #5 Sharma's poster #6 Scan your options—not your back
Sharma, Traeger, O'Keeffe, et al. (2021)	Australia	*Study objective* To evaluate the effects of information format on intentions to request diagnostic imaging for non‐specific low back pain in members of the public. *Participant involvement* To provide feedback on the readability, content, and usefulness of the leaflet.	No information	*People living with low back pain* *n* = 4 *Healthcare professionals* *n* = 4	*Three PEMs*: NSW agency for clinical innovation leaflet Back scan for low back pain Scan your options—not your back
Sharma, Traeger, Tcharkhedian, Middleton, et al. (2021)	Australia	*Study objective* To determine the effect of a waiting room communication strategy, designed to raise awareness of potential harms of unnecessary imaging, on lumbar imaging rates in the emergency department. *No participant involvement*	No information	No information	*Five PEMs*: Sharma's poster #7 Sharma's poster #8 Sharma's poster #9 Sharma's poster #10 Scan your options—not your back
Traeger et al. (2020)	Australia	*Study objective* To explore general practitioner and patient views of three communication tools (overdiagnosis leaflet, dialogue sheet and ‘wait‐and‐ see’ note) to support delayed prescribing of diagnostic imaging. *No participant involvement*	Acute	No information	Scan your options—not your back
de Campos et al. (2020)	Australia	*Study objective* What is the effect of a McKenzie‐based self‐management exercise and education programme on the risk of recurrence of low back pain and on the impact of low back pain? *No participant involvement*	Recent recovery (within the past 6 months)	No information	Managing back pain—Get back on track
Hodges et al. (2020)	Australia	*Study objective* To describe the multistep process undertaken to develop the MyBackPain website and provide an extensive evaluation of its impact. *Participant involvement* To identify consumer needs (step not reported in this article) To identify key messages (delphi process) To develop a frequently asked questions (focus groups) To review and refine the treatment summaries To test the website	Subacute and chronic	To identify key messages A multidisciplinary panel of experts and people living with low back pain To develop a FAQs People living with low back pain To review and refine the summaries *n* = 28 people living with low back pain *n* = 7 healthcare professionals working with people likely to have low health literacy To test the website *n* = 10 people living with low back pain *n* = 4 experts	MyBackPain
Yeh et al. (2020)	United States of America	*Study objective* To test auricular point acupressure as a non‐invasive, nonpharmacological self‐management strategy to manage chronic low back pain and to address current shortcomings of chronic low back pain treatment. *No participant involvement*	Chronic	No information	Back pain
Garofoli et al. (2019)	France	*Study objective* To evaluate the feasibility of such an intervention (i.e., a short multidisciplinary education and exercise therapy programme). *No participant involvement*	Subacute and chronic	No information	Five PEMs: Low back pain education & rehabilitation Our back at work Chronic low back pain and physical activity The back in everyday life Non‐pharmacologic management of chronic pain
Gardner et al. (2019)	Australia	*Study objective* To compare the clinical effectiveness and healthcare use of a patient‐led goal setting approach (intervention) with simple advice to exercise (control) over 12 months. *No participant involvement*	Chronic	No information	Low back pain, participant handbook
Jenkins et al. (2018)	Australia	*Study objective* To develop an implementation intervention aiming to reduce non‐indicated imaging for low back pain, by targeting both general medical practitioner general practitioners and patient barriers concurrently. *Participant involvement* To review the clinical resource To provide feedback on barriers and facilitators to implementing the clinical resource	Acute and chronic	To review the clinical resource *n* = 5 experts, including radiologists, rheumatologists, and general practitioners To provide feedback on barriers and facilitators *People living with low back pain* *n* = 10 5 females (50%) Mean age: 41.4 (range from 30 to 65) Information on education level and cultural background *General practitioners* *n* = 10 general practitioners 6 females (60%) Information on practice setting	Understanding my low back pain and whether I need imaging
Mansouri and Kostur (2018)	France	*Study objective* To observe, through a preliminary study, responses regarding the clinical relevance of pain neurophysiology education on pain intensity and the impact of low back pain on daily activities in individuals with chronic low back pain, and to assess whether its illustration through a brochure is an appropriate tool. *Participant involvement* To respond to a questionnaire to identify knowledge gaps and misconceptions about pain neurophysiology, which informed the development of the brochure. To review the content of the brochure.	Chronic	To identify knowledge gaps *n* = 42 people living with low back pain To review the content *n* = 12, including 10 pain specialists and 2 physiotherapists	Que se passe‐t‐il dans mon corps quand j'ai mal ?
Patel et al. (2014)	United Kingdom	*Study objective* To pilot a decision support package to help people choose between low back pain treatments. *No participant involvement*	No information	No information	IMPACT LBP (IMproving PAtient choice in treatment of LBP)
Saper et al. (2014)	United States of America	*Study objectives* In the 12‐week treatment phase, compare the effectiveness between a structured protocol of one yoga class per week, an individually‐delivered structured physical therapy protocol established around evidence‐based clinical guidelines, and an educational book on self‐care for chronic low back pain. In the 40‐week maintenance phase, compare the effectiveness between patients participating in a structured yoga maintenance programme, a structured physical therapy maintenance programme, or no structured maintenance programme. Determine the cost‐effectiveness of yoga, physical therapy, and education for adults with chronic low back pain at 12 weeks, 6 months, and 1 year from three perspectives: Society, third‐party payer, and the participant. *No participant involvement*	Chronic	No information	*Four PEMs*: Back to health: How to safely resume your normal activities Back to health: Effective self‐care Back to health: Physical activity and exercise Back to health: Everyday insights for better living

Abbreviations: FAQs, frequently asked questions; LBP, low back pain; NSW, New South Wales; PEMs, patient education materials; SD, standard deviation.

Four studies (17.4%) reported the objective to develop a PEM. Seventeen studies (73.9%) employed a single PEM, whereas six studies (26.1%) used multiple PEMs, with the number of PEMs ranging from three to seven. Four studies (17.4%) used the Scan your options, not your back and two studies (8.7%) employed the Understanding low back pain and whether I need imaging.

Stakeholder involvement in the development or pilot testing of patient education materials was reported in eight studies (34.8%). Their involvement varied, ranging from manuscript review to a more active role in the co‐design of the PEM. Among the eight studies, engagement included individuals living with LBP (*n* = 8/8, 100%), clinicians (*n* = 5/8, 62.5%), experts (*n* = 2/8, 25.0%), and carers (*n* = 1/8, 12.5%). Three (37.5%) studies provided stakeholder characteristics. Two of them reported participants' health literacy levels. From the PROGRESS + framework, information on education level, sex/gender, and age were available in the three studies, while ethnicity, culture, opioid consumption, and practice setting were available in one study (12.5%). No study involving people in the development of the education material reported information on all characteristics outlined in the PROGRESS + framework.

### 3.3 Characteristics of the Patient Education Materials

The patient education material characteristics are presented in Table 4. We identified 41 patient education materials, comprising 12 posters (29.3%), eight booklets (19.5%), eight leaflets (19.5%), five slideshows (12.2%), four newsletters (9.7%), three websites (7.3%), and one video (2.4%). The number of pages in the booklets ranged from 7 to 27. Out of the 41 PEMs, 39 (95.1%) were exclusively available in English, one (2.4%) in French, and one (2.4%) in multilingual. Six studies (14.6%) required a fee for access.

**TABLE 4:** Characteristics of the patient education materials.

Name of the patient education material	Format	Language	Length of the PEM	Reported readability	Main topics	Free access to the PEM
Understanding and coping with pain (Tomás‐Rodríguez et al. 2024)	Slideshow	English	37 slides	No information	What do you think pain is?/What is it for?/Why do we feel pain?/Chronic pain solutions	No
Back book (Marins et al. 2023)	Booklet	English	14 pages	No information	Backs facts/Causes/Rest or active exercises?/Exercise is good for you, staying active/How to stay active/When to see your doctor?/It's your back	Yes
MyRelief (Marley et al. 2023)	Website	English	1h55	No information	Understanding persistent low back pain/Why physical activity and exercise are important/Why mental health and psychology are important/Sleep, diet and nutrition/Managing LBP in your workplace/Communication with health services/Common questions	Yes, for the 1h55 tutorial
Reducing my opioids (medicine to relieve pain) (Marcelo et al. 2023)	Booklet	English	8 pages	Flesch‐Kincaid: 8.6	What is chronic musculoskeletal pain?/What are opioids?/Why should I reduce or stop my opioid?/What side effects can I experience while taking opioids?/How do I stop taking my opioid?/What should I watch for when coming off my opioid?/What non‐drug options can I do to manage my pain?/What should I do if I continue to feel worse?/Who do I contact to reduce or stop my opioid?	Yes
Ergonomic brochure: Instructions for daily routine (Agre & Agrawal 2023)	Leaflet	English	2 pages	No information	Use support while standing/Appropriate footwear/While working for assignment/Maintain good standing posture/Lift with care/Designing of working station/When correcting assignment/When approaching kids/When on desk with tiny toys/When playing with kids/Sit instead/Sit straight	No
Know what you are taking (Patterson et al. 2022)	Booklet	English	12 pages	No information	Did you know?/The downward spiral of pain/The upward spiral of exercising/Relieving low back pain without paracetamol/Pace yourself: Exercise and strength/How to create an activity programme/Activity diary/Testimony/4 questions to ask your health care professional	Yes
How can back pain affect you? (Webber et al. 2022)	Leaflet	English	2 pages	No information	Information on back pain/Consequences of back pain	Yes
Managing low back pain (Zadro et al. 2022)	Leaflet	English	2 pages	No information	Serious conditions are very rare/Remember that most people don't need scans/Keep moving—it's the best thing to do/Use simple pain relief strategies/Follow up with your local health professional/Remind yourself that most people get better with time	Yes
Management of low back pain—right scenario versus wrong scenario (Diniz et al. 2022)	Video	Portuguese, English	6′48	No information	A story of a character who developed low back pain after carrying a box in the garage/Then, two possible scenarios are contrasted during the story, one management strategy that follows clinical practice guideline recommendations versus one based on low‐value care.	Yes
Low back pain: Assessment and imaging (Diniz et al. 2022)	Poster	English	1 page	No information	Low back pain definition/What are the important aspects to consider in the assessment?/Are image exams necessary?	No
Low back pain: Diagnosis and treatment (Diniz et al. 2022)	Leaflet	English	2 pages	No information	Diagnosis and treatment of low back pain	No
Low back pain: General treatments (Diniz et al. 2022)	Poster	English	1 page	No information	Myths and truths about treatments	No
Understanding low back pain and whether I need imaging (Simula et al. 2021; Jenkins et al. 2018)	Booklet	English	7 pages (Simula et al. 2021), 8 pages (Jenkins et al., 2018)	No information	Do I need imaging or further investigations?/All about low back pain/Why isn't imaging needed?/What can I do to help decrease my low back pain?/What is my low back pain management plan?/Further information on low back pain	Yes
Sharma's poster #1 (Sharma, Traeger, Tcharkhedian, et al. 2021)	Poster	English	1 page	No information	Radiation from scan increase your chance of cancer	Yes
Sharma's poster #2 (Sharma, Traeger, Tcharkhedian, et al. 2021)	Poster	English	1 page	No information	Back scan may lead to dangerous and unnecessary treatments	Yes
Sharma's poster #3 (Sharma, Traeger, Tcharkhedian, et al. 2021)	Poster	English	1 page	No information	Over 2/3 of people who have a back scan will get a false alarm	Yes
Sharma's poster #4 (Sharma, Traeger, Tcharkhedian, et al. 2021)	Poster	English	1 page	No information	Back scan can't heal‐they can harm	Yes
Sharma's poster #5 (Sharma, Traeger, Tcharkhedian, et al. 2021)	Poster	English	1 page	No information	Most people won't benefit from having a scan. It won't find the cause of the pain, and leads to harmful, ineffective treatment	Yes
Sharma's poster #6 (Sharma, Traeger, Tcharkhedian, et al. 2021)	Poster	English	1 page	No information	Ask your doctor‐ do I need this test	Yes
Scan your options—not your back (Sharma, Traeger, O'Keeffe, et al. 2021; Sharma, Traeger, Tcharkhedian, et al. 2021; Sharma, Traeger, Tcharkhedian, Middleton, et al. 2021; Traeger et al., 2020)	Leaflet	English	2 pages	Grade 7 (Sharma, Traeger, O'Keeffe, et al. 2021)	What are back scans?/Back scans for low back pain/Get back to better	Yes (Sharma, Traeger, Tcharkhedian, et al. 2021; Sharma, Traeger, Tcharkhedian, Middleton, et al. 2021; Traeger et al., 2020)
NSW agency for clinical innovation leaflet (Sharma, Traeger, O'Keeffe, et al. 2021)	Leaflet	English	2 pages	Grade 7	Understanding low back pain/tips for a rapid recovery/Exercise	No
Back scan for low back pain (Sharma, Traeger, O'Keeffe, et al. 2021)	Leaflet	English	2 pages	Grade 7	What are back scans?/Back scan for low back pain/Get back to better	No
Sharma's poster #7 (Sharma, Traeger, Tcharkhedian, Middleton, et al. 2021)	Poster	English	1 page	No information	Back scan can cause harm	Yes
Sharma's poster #8 (Sharma, Traeger, Tcharkhedian, Middleton, et al. 2021)	Poster	English	1 page	No information	Many people who have a back scan will get a false alarm	Yes
Sharma's poster #9 (Sharma, Traeger, Tcharkhedian, Middleton, et al. 2021)	Poster	English	1 page	No information	False alarms on back scans can lead to ineffective surgery	Yes
Sharma's poster #10 (Sharma, Traeger, Tcharkhedian, Middleton, et al. 2021)	Poster	English	1 page	No information	Back scans rarely find the cause of back pain. Before having the test, ask your doctor what the alternatives are for you	Yes
Managing back pain—Get back on track (de Campos et al. 2020)	Booklet	English	25 pages	No information	About this guide/What is back pain?/Causes of back pain/Diagnosing chronic back pain/Why do I need a back pain action plan?/Managing back pain/Preventing back pain/The back pain action plan/More information	Yes
MyBackPain (Hodges et al. 2020)	Website	English	10 pages, 7 videos from 2' to 10'	No information	About back pain/Do it yourself tips/Treatments/health care professionals/Test your knowledge/For family and friends/videos	Yes
Back pain (Yeh et al. 2020)	Website	English, Spanish, Vietnamese, Korean, Chinese	3 pages	No information	Overview, symptoms and causes/Diagnosis, treatment and step to take/Research and resources	Yes
Low back pain education & rehabilitation (Garofoli et al. 2019)	Slideshow	English	14 slides	No information	Low back pain/Public health/Pathway of pain/The importance of maintaining professional activity/Factors involved in progression to chronicity of low back pain/Cycle of pain/What to do/Objectives/Helpful strategies	Yes
Our back at work (Garofoli et al. 2019)	Slideshow	English	7 slides	No information	We were made to move!/Spine: Made for movement/Non‐specific low back pain/Avoid low back pain?/What to do to avoid/treat low back pain?	Yes
Chronic low back pain and physical activity (Garofoli et al. 2019)	Slideshow	English	6 slides	No information	Benefits of physical activity/WHO recommendations/Examples of physical activities/Physical activity and diseases	Yes
The back in everyday life (Garofoli et al., 2019)	Booklet	English	8 pages	No information	Anatomy/Find relief: Relaxation, heat, Swiss ball…/Automatic postural principles/Loading/Some ‘comfort’ tips for everyday life	Yes
Non‐pharmacologic management of chronic pain (Garofoli et al., 2019)	Slideshow	English	11 slides	No information	Definition/Consequences of chronic pain/pain management/Aggravating factors of chronic pain/How to recognise depression/The vicious cycle of chronic pain	Yes
Low back pain, participant handbook (Gardner et al. 2019)	Booklet	English	23 pages	No information	Chronic low back pain, An overview: Acute versus chronic/Causes of low back pain; rest or maintain normal activity?/Managing a ‘flare up’ of low back pain/Serious conditions—10 APA tips for back care—self‐management of low back pain: Achieving optimal self‐management of chronic low back pain/Optimising your self‐management of chronic low back pain/Setting goals/Setting your goals for managing your chronic low back pain	No
Que se passe‐t‐il dans mon corps quand j'ai mal ? (Mansouri & Kostur 2018)	Leaflet	French	2 pages	No information	How does pain arise?/The different types of pain/Its various components/Pain modulation/How can I modify my pain?/Have you understood everything?	No
IMPACT LBP (IMproving PAtient choice in treatment of LBP) (Patel et al. 2014)	Booklet	English	27 pages	No information	About this booklet/Welcome/Step 1: What treatment options do I have?/What matters to you when choosing treatment?/What else do I need to know to make a decision? What next?	Yes
Back to health: How to safely resume your normal activities (Saper et al. 2014)	Newsletter	English	1 page	Grade 6	What should I do if I'm having severe pain?/What if I am having a severe flare‐up and it is too painful to move?/What should I be doing as my severe pain gets better?/Because of my back pain, should I be doing fewer activities?/What if an activity increases my pain throughout the day?/When I have a flare‐up, I like to stay in bed or frozen in one position. So, I should push through the pain?/After a flare‐up, should I do as little as possible?	Yes
Back to health: Effective self‐care (Saper et al. 2014)	Newsletter	English	3 pages	Grade 6	4 steps to making a plan of action/The three R's for managing flare‐ups/How to talk to your doctor about back pain/Tips for taking over‐the‐counter medicine/Managing flare‐ups and emergencies/Physical methods for back pain (some exercises require a commercial book)/Mind‐body techniques (some exercises require a commercial book)/Using deep breathing as a relaxation tool/Tips for coping with depression.	Yes
Back to health: Physical activity and exercise (Saper et al. 2014)	Newsletter	English	1 page	Grade 6	Four basics to good mechanics (some exercises require a commercial book)/Benefits of stretching/Exercises for building strength and endurance (all exercises require a commercial book)/tips for increasing strength and endurance, six steps for finding time for exercise. (Some exercises require a commercial book).	Yes
Back to health: Everyday insights for better living (Saper et al. 2014)	Newsletter	English	1 page	Grade 6	Everyday insights for better living: How to re‐establish a normal sleeping pattern/How to prevent future sleep problems/How to avoid problems in your relationships/Tips for workers with back pain/The American chronic pain Association's 10 steps for dealing with pain.	Yes

Abbreviations: APA, Australian Physiotherapy Association; LBP, low back pain; NSW, New South Wales; PEM, patient education material; WHO, World Health Organisation.

Eight PEMs (19.5%) were assessed for readability. One PEM was evaluated using the Flesch‐Kincaid Grade Level test, achieving a score of 8.6. The remaining PEMs reported readability levels corresponding to grades 6 or 7 but did not specify the readability assessment method used.

The main topics addressed in these 41 PEMs were: (1) understanding pain, including definitions, distinguishing between acute and chronic forms, and explained pain's biological functions; (2) imaging and diagnostic tests, discussing necessity and risks associated with imaging; (3) physical activity and exercise, presenting recommendations, benefits, and exercise plans; (4) non‐pharmacological pain management strategies, including ergonomics, posture, relaxation, and pacing techniques; (5) medication management, specifically opioid use, reduction, and side effects; (6) psychological factors and mental health considerations, particularly emphasising coping skills and addressing emotional wellbeing; (7) self‐care and coping strategies, including strategies to manage flare‐ups and promote wellbeing; (8) communication and decision‐making with healthcare professionals; (9) ergonomic guidance for workplace and daily activities; (10) sleep, nutrition, and lifestyle recommendations; and (11) clarification of myths and misconceptions related to pain management.

### 3.4 Alignment Between PEMs Content and Information Needs

We present the results of the consensus approach regarding the alignment between PEMs content and information needs in Table 5. Out of the 41 PEMs, one (2.4%) addressed all information needs of people living with non‐specific low back pain. Five PEMs (12.2%) met at least the three quarters of the information needs. The most met need in the identified PEMs was information on treatment options (*n* = 27, 65.9%), followed by information on imaging (*n* = 25, 61.0%), on self‐management strategies (*n* = 23, 56.1%), and general information on LBP (*n* = 22, 53.7%). Information on prognosis (*n* = 7, 17.1%) and on flares and prevention (*n* = 7, 17.1%) were the less addressed needs.

**TABLE 5:** Alignment between patient education materials content and information needs of people living with low back pain it addresses.

Name of the PEM	General information related to LBP	Diagnosis and cause	Needs for imaging	Prognosis	Flares and preventive measures	LBP management	Self‐management strategies	Support services
Understanding and coping with pain						X		
Back book	X	X	X			X	X	X
MyRelief	X	X	X			X	X	X
Reducing my opioids (medicine to relieve pain)	X					X	X	X
Ergonomic brochure: Instructions for daily routine							X	
Know what you are taking	X					X	X	
How can back pain affect you?	X							
Managing low back pain	X		X	X		X	X	X
Management of low back pain—right scenario versus wrong scenario	X	X	X	X		X		
Low back pain: Assessment and imaging	X		X			X		
Low back pain—Diagnosis and treatment	X	X	X	X		X		
Low back pain: General treatments	X		X			X	X	
Understanding low back pain and why I need imaging	X	X	X			X	X	X
Sharma's poster #1			X					
Sharma's poster #2			X					
Sharma's poster #3			X					
Sharma's poster #4			X					
Sharma's poster #5			X					
Sharma's poster #6			X					
Scan your options—not your back			X			X	X	
NSW agency for clinical innovation leaflet	X	X	X	X		X	X	X
Back scan for low back pain			X			X	X	X
Sharma's poster #7			X					
Sharma's poster #8			X					
Sharma's poster #9			X					
Sharma's poster #10			X					
Managing back pain—Get back on track	X	X	X	X	X	X	X	X
MyBackPain	X		X		X	X	X	X
Back pain	X	X	X		X	X	X	X
Low back pain education & rehabilitation	X			X		X		
Our back at work	X				X		X	
Chronic low back pain and physical activity						X	X	
The back in everyday life	X					X	X	
Non‐pharmacologic management of chronic pain	X					X	X	
Low back pain, participant handbook	X	X	X		X	X	X	X
Que se passe‐t‐il dans mon corps quand j'ai mal?	X					X		
IMPACT LBP (IMproving PAtient choice in treatment of LBP)	X	X		X		X		X
Back to health: How to safely resume your normal activities					X	X	X	
Back to health: Effective self‐care					X	X	X	
Back to health: Physical activity and exercise						X	X	
Back to health: Everyday insights for better living							X	

Abbreviations: LBP, low back pain; NSW, New South Wales; PEM, patient education material.

## 4 Discussion

In this scoping review, we identified 41 accessible patient education materials (PEMs) addressing 11 key topics relevant to individuals living with non‐specific low back pain (LBP). Our analysis indicated that only one PEM comprehensively addressed all identified information needs for this population. Eight studies involved stakeholders in the development of PEMs, and stakeholder characteristics were seldom reported. Readability assessments of the PEMs were infrequently conducted, and when performed, the assessment methods were inadequately described. This led us to make four observations.

Limited access to free patient education materials is a major barrier to integrating evidence‐based education into clinical practice. In our review, 467 citations were excluded due to restricted access, and six of included articles required paid access. These financial and logistical barriers force clinicians to purchase multiple scientific articles without assessing their quality and relevance beforehand. As a result, they may waste resources on ineffective or misleading materials. For example, some restricted‐access materials recommend keeping the back straight for spinal protection despite no supporting evidence (Saraceni et al. 2020; Slater et al. 2019). Paying for low‐value content increases inefficiencies by requiring additional effort to identify reliable resources. It may also discourage future investment in patient education, further limiting access to high‐quality information. Limited accessibility also affects research. Because researchers rarely share data from their studies (Gabelica et al. 2022; Watson 2022), new educational resources are often developed independently rather than refined or validated based on existing ones. This redundancy leads to an overabundance of diverse materials, making it difficult to synthesise findings in systematic reviews and meta‐analyses. As a result, different educational interventions are often grouped under a single ‘education’ label, weakening the reliability of synthesised evidence. Ultimately, restricted access to patient education materials deprives individuals with non‐specific low back pain of a critical component of their care. Addressing these barriers is essential for improving clinical practice and research integrity.

Future PEMs should integrate stakeholder involvement and clearly report the level of engagement. We found that only one‐third of the included studies explicitly reported engaging stakeholders in the development or pilot phase. Failure to meaningfully involve patients in PEM development risk misalignment between educational content and the actual priorities of people with low back pain. French et al. highlighted a critical discrepancy: while experts prioritise messages avoiding unnecessary imaging, patients rank this topic among their lowest concerns (French et al. 2019). This misalignment is reflected in our results, where three‐quarters of the identified PEMs emphasised this issue despite limited patient interest, suggesting a dominant influence of expert‐driven perspectives over person‐centred priorities. Among the eight studies reporting stakeholder engagement, the level of involvement ranged from passive consultation—such as reviewing content—to active collaboration, where stakeholders co‐designed the PEMs (Gibbins and Lo 2022). Passive consultation alone fails to capture what truly matters to patients; meaningful involvement—at least at the level of collaboration—is necessary to ensure that patient perspectives genuinely inform the content and delivery of interventions (Greenhalgh et al. 2019; National Institute for Health Research 2013). Greater transparency in reporting—not only the presence but also the extent and impact of stakeholder involvement—is essential for assessing its role in producing PEMs that are both evidence‐based and responsive to patient needs.

Future PEMs should systematically report the characteristics of stakeholders involved in their development. In our review, only three studies provided such information, and even then, the characteristics reported were limited when assessed with the PROGRESS‐Plus framework. This lack of reporting raises concerns about the extent to which development processes reflect the diversity of intended users and the broader relevance and generalisability of the resulting interventions (Clark et al. 2019; National Academies of Sciences et al. 2022). The overrepresentation of WEIRD (Western, Educated, Industrialised, Rich, and Democratic) populations in health research is well documented (Henrich et al. 2010). PEMs developed within this context tend to align with dominant cultural norms, which could overlook or misrepresent the needs, values, and lived experiences of other groups. Many materials adopt a biomedical framing that emphasises individual responsibility and adherence, often overlooking the determinants of health (Roy 2008). This approach can conflict with the relational, spiritual, or community‐based models of illness prevalent in Indigenous, immigrant, or racialised populations (Asamoah et al. 2023; Haynes et al. 2021). When these perspectives are absent from the development process, PEMs risk being ineffective, alienating, or even stigmatising. Health literacy is also rarely reported, despite information that literacy and education levels influence patients' ability to engage with health information (Oosterhaven et al. 2023). Oosterhaven et al. showed that poorly adapted materials can lead to difficulties in understanding the message, negative evaluations of the information, and an inability to apply it in daily life (Oosterhaven et al. 2023). These risks reflect deeper structural issues in how PEMs are often developed, particularly the lack of attention to equity, representation, and the diversity of patient experiences. To ensure PEMs are effective, equitable, and trustworthy, development processes must include a broad range of stakeholders reflecting the target population.

Our findings offer clinicians a range of tools to support personalised and practical integration of patient education into routine care. Only one PEM identified addressed all identified information needs, highlighting the gap in comprehensive educational resources for people living with low back pain. Nevertheless, clinicians can still use a combination of targeted PEMs to tailor education to individual needs. This personalised approach may help ensure that the information provided is relevant and meaningful. However, clinical integration remains challenging: most PEMs are available only in English, their readability is rarely assessed, and their length can risk overwhelming individuals with excessive information (Askin 2017; Crane 1997; Wang and Voss 2022). A growing number of studies now explore the potential of large language models, such as ChatGPT, Claude, and Gemini, to generate simplified and accessible health education content (Nasra et al. 2025; Thirunavukarasu et al. 2023). These models can produce coherent, human‐like responses and may support clinicians by delivering personalised, readable content (Khurana et al. 2023). Recent evaluations have shown high user satisfaction and improved readability (Nasra et al. 2025), and models like ChatGPT‐4 have demonstrated high accuracy in addressing common questions about chronic low back pain (Liu et al. 2024). Nonetheless, concerns remain regarding the consistency and specificity of responses, particularly for treatment‐related information, and the risk of outdated or overly generic content due to reliance on static training data (Liu et al. 2024). Ongoing evaluation and continuous updating of these tools will be essential to ensure their safe and effective use in clinical education.

## 5 Limitations

This scoping review has several limitations. First, we only included PEMs that were freely accessible through the scientific literature. Numerous PEMs were excluded due to restricted access or non‐functional links. While this choice may have limited the comprehensiveness of our review, it allowed us to highlight a critical issue: the limited accessibility of PEMs is a significant barrier to their integration into clinical practice. By focussing on accessible materials, we aimed to reflect the real‐world availability of educational resources for clinicians and patients and to expose structural barriers hindering their use.

Second, we did not involve an information specialist in the development of our search strategy. However, to enhance its rigour, the search was peer‐reviewed by an independent assessor using the PRESS checklist, and we conducted additional handsearching of recent reviews and backward‐forward citation tracking to reduce the risk of missing relevant studies.

Third, the categorisation of information needs and the mapping of PEM content were based on consensus among reviewers using the framework proposed by Lim et al. Due to the lack of precise definitions for each need, an element of subjectivity may have been introduced in our analysis, as reviewers had to interpret categories. However, to limit potential misinterpretation due to overlapping categories, we merged some needs, which facilitated consensus but may have led to a loss of nuance that could be important for adequately capturing the full range of people living with low back pain needs.

Fourth, we did not assess the quality of the content within the included PEMs. As noted in the discussion, some materials available in the literature recommend outdated or unsupported practices. Clinicians should therefore critically appraise the quality and relevance of any PEM identified in this review before integrating it into clinical practice.

## 6 Conclusion

We identified 41 accessible patient education materials (PEMs) for people living with non‐specific low back pain. Only one addressed all identified information needs of this population. Stakeholder involvement in the development or pilot testing of these materials was infrequent and varied between passive consultation and more active collaboration. When stakeholders were involved, their characteristics were seldom reported, limiting the ability to assess whether diverse perspectives were meaningfully included. Readability assessments were also rarely conducted, potentially resulting in materials that are unsuitable for individuals with lower education level and health literacy. To improve the relevance and usability of PEMs, both active stakeholder involvement and open access to materials are essential. Future development should prioritise co‐design approaches, transparency in reporting participant characteristics, and readability optimisation to ensure that educational resources truly address people's information needs in diverse populations.

## Author Contributions

**Chloé Debonne:** conceptualisation, investigation, methodology, visualisation, writing – original draft. **Axel Houdart:** conceptualisation, investigation, methodology, visualisation, writing – original draft. **Chloé Cachinho:** conceptualisation, investigation, methodology, writing – review and editing. **Alexy Ouvrier‐Neyret:** conceptualisation, investigation, methodology, writing – review and editing. **Thomas Gérard:** conceptualisation, investigation, methodology, writing – review and editing. **Valentin Vaillant:** methodology, writing – review and editing. **Yannick Tousignant‐Laflamme:** methodology, writing – review and editing. **Marie‐Pierre Gagnon:** methodology, writing – review and editing. **Maxime Sasseville:** methodology, writing – review and editing. **Simon Décary:** methodology, writing – review and editing. **Florian Naye:** conceptualisation, investigation, methodology, visualisation, writing – original draft.

## Conflicts of Interest

The authors declare no conflicts of interest.

## Supporting information

Supporting Information S1

## Data Availability

The authors have nothing to report.
