# Supplementary material for: Accessible Patient Education Materials for Low Back Pain Rarely Meet People's Information Needs: A Scoping Review
Source: Musculoskeletal Care. 2025 May 30;23(2):e70130. doi: 10.1002/msc.70130 (PMC12124170; doi:10.1002/msc.70130)
Supplement: Supplementary file 1 — Supporting Information S1 [file MSC-23-e70130-s001.docx]

**Appendix 1: Search strategy**

| Databases | # | Search strategy | Results |
| --- | --- | --- | --- |
| PubMed | 1 | “consumer health” [title/abstract] OR communicat* [title/abstract] OR educat* [title/abstract] OR informat* [title/abstract] OR handout [title/abstract] OR teach* [title/abstract] OR workshop [title/abstract] OR “training program*” [title/abstract] OR “literacy program*” [title/abstract] | 2 960 307 |
|  | 2 | “low back pain” [title] OR “lower back pain” [title] OR “low back ache” [title] OR “low backache” [title] OR “lower backache” [title] | 19 123 |
|  | 3 | #1 AND #2 | 3 039 |
| Scopus | 1 | TITLE-ABS-KEY (“consumer health” OR “communicat*” OR “educat*” OR “informat*” OR “handout” OR “teach*” OR “workshop” OR “training program*” OR “literacy program*”) | 12 239 084 |
|  | 2 | TITLE (“low back pain” OR “lower back pain” OR “low back ache” OR “low backache” OR “lower backache”) | 23 927 |
|  | 3 | #1 AND #2 | 4 446 |
| Cinahl | 1 | “consumer health” [title] OR communicat* [title] OR educat* [title] OR informat* [title] OR handout [title] OR teach* [title] OR workshop [title] OR “training program*” [title] OR “literacy program*” [title] | 314 424 |
|  | 2 | “consumer health” [abstract] OR communicat* [abstract] OR educat* [abstract] OR informat* [abstract] OR handout [abstract] OR teach* [abstract] OR workshop [abstract] OR “training program*” [abstract] OR “literacy program*” [abstract] | 845 272 |
|  | 3 | “low back pain” [title] OR “lower back pain” [title] OR “low back ache” [title] OR “low backache” [title] OR “lower backache” [title] | 14 425 |
|  | 4 | #1 OR #2 | 1 030 588 |
|  | 5 | #3 and #4 | 2 117 |
